# Clinical application of targeted tumour sequencing tests for detecting *ERBB2* amplification and optimizing anti-HER2 therapy in gastric cancer

**DOI:** 10.1186/s12885-024-12482-5

**Published:** 2024-06-11

**Authors:** Hiroshi Ichikawa, Kenji Usui, Masaki Aizawa, Yoshifumi Shimada, Yusuke Muneoka, Yosuke Kano, Mika Sugai, Kazuki Moro, Yuki Hirose, Kohei Miura, Jun Sakata, Hiroshi Yabusaki, Satoru Nakagawa, Takashi Kawasaki, Hajime Umezu, Shujiro Okuda, Toshifumi Wakai

**Affiliations:** 1 Division of Digestive and General Surgery, Niigata University Graduate School of Medical and Dental Sciences, 1-757 Asahimachi-dori, Chuo-ku, Niigata City, Niigata 951-8510 Japan; 2 Department of Gastroenterological Surgery, Niigata Cancer Center Hospital, 2-15-3 Kawagishi-cho, Chuo-ku, Niigata City, Niigata 951-8566 Japan; 3 Division of Medical Technology, Niigata University Graduate School of Health Sciences, 2-746 Asahimachi-Dori, Chuo-ku, Niigata City, Niigata 951-8518 Japan; 4 Department of Pathology, Niigata Cancer Center Hospital, 2-15-3 Kawagishi-cho, Chuo-ku, Niigata City, Niigata 951-8566 Japan; 5 Division of Pathology, Niigata University Medical and Dental Hospital, 1-754 Asahimachi-dori, Chuo-ku, Niigata City, Niigata 951-8520 Japan; 6 Division of Bioinformatics, Niigata University Graduate School of Medical and Dental Sciences, 2−5274, Gakkocho-dori, Chuo-ku, Niigata City, Niigata 951-8514 Japan

**Keywords:** Gastric cancer, HER2, *ERBB2*, Targeted tumour sequencing test, Trastuzumab

## Abstract

**Background:**

Evaluation of human epidermal growth factor receptor 2 (HER2) overexpression caused by erb-b2 receptor tyrosine kinase 2 (*ERBB2*) amplification (AMP) by immunohistochemistry (IHC) and fluorescence in situ hybridization (FISH) is essential for treating unresectable metastatic gastric cancer (GC). A targeted tumour sequencing test enables comprehensive assessment of alterations in cancer-related genes, including *ERBB2*. This study aimed to evaluate the concordance between the targeted tumour sequencing test and IHC/FISH for detecting HER2-positive GC and to clarify the significance of *ERBB2* AMP and concomitant genetic alterations in HER2 downstream pathways (DPs) in anti-HER2 therapy for unresectable metastatic GC patients.

**Methods:**

*ERBB2* copy number alteration (CNA) was examined via a targeted tumour sequencing test in 152 formalin-fixed paraffin-embedded (FFPE) GC tissues. *ERBB2* CNA was compared to HER2 status evaluated by IHC/FISH in FFPE block sections, which were identical to those subjected to the targeted tumour sequencing test. Treatment outcomes of anti-HER2 therapy in 11 patients with unresectable metastatic GC was evaluated.

**Results:**

*ERBB2* AMP (≥ 2.5-fold change) was detected by the targeted tumour sequencing test in 15 patients (9.9%), and HER2 positivity (IHC 3 + or IHC 2+/FISH positive) was detected in 21 patients (13.8%). The overall percent agreement, positive percent agreement, negative percent agreement and Cohen’s kappa between *ERBB2* CNA and HER2 status were 94.7%, 66.7%, 99.2% and 0.75, respectively. Progression-free survival for trastuzumab therapy in patients with *ERBB2* AMP was significantly longer than that in patients with no *ERBB2* AMP detected by the targeted tumour sequencing test (median 14 months vs. 4 months, *P* = 0.007). Treatment response to trastuzumab therapy was reduced in patients with *ERBB2* AMP and concomitant CNAs of genes in HER2 DPs. One patient with *ERBB2* AMP and concomitant CNAs of genes in HER2 DPs achieved a durable response to trastuzumab deruxtecan as fourth-line therapy.

**Conclusions:**

A targeted tumour sequencing test is a reliable modality for identifying HER2-positive GC. *ERBB2* AMP and concomitant genetic alterations detected through the targeted tumour sequencing test are potential indicators of treatment response to trastuzumab therapy. The targeted tumour sequencing test has emerged as a plausible candidate for companion diagnostics to determine indications for anti-HER2 therapy in the era of precision medicine for GC.

**Supplementary Information:**

The online version contains supplementary material available at 10.1186/s12885-024-12482-5.

## Background

Despite recent advancements in diagnostic and therapeutic modalities, gastric cancer (GC) remains the third leading cause of cancer death worldwide [1]. Prognosis of patients with endoscopically or surgically resectable disease is relatively acceptable [2–4]. However, unresectable metastatic disease is notorious for its unfavourable outcome, with a median overall survival time of only 17 to 18 months [5, 6]. Many large-scale phase III clinical trials to develop molecular targeted therapies have been conducted [7]. Trastuzumab is the initially approved molecular targeted therapy with proven efficacy as first-line treatment for human epidermal growth factor receptor 2-positive (HER2-positive) unresectable metastatic GC [8]. Thus, it is essential to evaluate HER2 overexpression by immunohistochemistry (IHC) and erb-b2 receptor tyrosine kinase 2 (*ERBB2*) amplification (AMP) by fluorescence in situ hybridization (FISH) when treating unresectable metastatic GC [9].

Genomic profiling using a targeted tumour sequencing test plays an important role in optimized treatment of cancer. It allows for comprehensive evaluation of genomic alterations, including substitutions, insertions and deletions (indels), copy number alterations (CNAs), and gene rearrangements in multiple genes, as well as genomic signatures, including microsatellite instability (MSI) and the tumour mutational burden (TMB), in a single test [10, 11]. Therefore, multiple therapeutic options can be offered, and genomic alterations related to treatment resistance can potentially be evaluated at the same time [11]. GC, in particular, is a cancer with a heterogeneous molecular background, which is thought to be the reason why few molecularly targeted therapies have been effective in clinical trials to date [12]. In general, genomic profiling of individual patients through the targeted tumour sequencing test will aid in optimization of molecularly targeted therapies for unresectable metastatic GC [13]. To this end, the concordance between *ERBB2* CNA detected by the targeted tumour sequencing test and HER2 status determined by IHC and FISH, which are companion diagnostics for trastuzumab therapy, needs to be elucidated. In addition, clarification of the association between genomic alterations in HER2 downstream pathways (DPs) and the efficacy of anti-HER2 therapy might be beneficial for optimizing molecular targeted therapy for GC.

In this study, we aimed to clarify the concordance between *ERBB2* CNA detected by a targeted tumour sequencing test and HER2 status evaluated by IHC and FISH using the genomic profile of GC in our previous study [14]. In addition, we evaluated the association between the degree of *ERBB2* AMP, as well as genetic alterations in HER2 DPs, and treatment response to anti-HER2 therapy for unresectable metastatic GC.

## Materials and methods

### Patients and clinicopathological background

We enrolled a total of 152 patients who underwent anticancer treatment for GC between 2009 and 2019 at Niigata University Medical and Dental Hospital or Niigata Cancer Center Hospital. Among these patients, 130 were included in our previous genomic sequencing study for GC using a targeted tumour sequencing test [14]. The clinicopathological features of the enrolled patients are summarized in Table 1. The pathological tumour stage of the GC patients was evaluated according to the 8th edition of the Union for International Cancer Control Tumour-Node-Metastasis (TNM) classification system [15]. There were 110 men and 42 women, with a median age (range) of 68 years (27–87 years). Gastrectomy and lymphadenectomy with curative intent were performed for 151 patients. Trastuzumab combined with chemotherapy was administered to 11 patients as first-line therapy for unresectable metastatic GC. Written informed consent for participation in this study was obtained from all subjects. This study was approved by the institutional review board of the Niigata University Medical and Dental Hospital (G2020-0038) and Niigata Cancer Center Hospital (2015-73) in accordance with the Helsinki Declaration and the Ethical Guidelines for Medical and Biological Research Involving Human Subjects in Japan [16].

**Table 1:** Summary of clinicopathological characteristics, *ERBB2* CNA and HER2 status (*N* = 152)

Characteristics	Number of patients (%)
Sex	
Male	110 (72.4)
Female	42 (27.6)
Age (years)	
Median (range)	68 (27–87)
Location	
GEJ or Cardia	27 (17.8)
Body or Fundus	58 (38.2)
Antrum	67 (44.1)
Lauren classification	
Intestinal	90 (59.2)
Diffuse	40 (26.3)
Mixed	9 (5.9)
Indeterminate	13 (8.6)
T classification*	
T1	4 (2.7)
T2	23 (15.1)
T3	69 (45.4)
T4	56 (36.8)
N classification*	
N0	41 (27.0)
N1	30 (19.7)
N2	23 (15.1)
N3	58 (38.2)
M classification*	
M0	123 (80.9)
M1	29 (19.1)
EBV infection	
Absent	148 (97.4)
Present	4 (2.6)
MSI status	
MSI-high	13 (8.6)
MSI-low or MSS	139 (97.4)
Gastrectomy	
Performed	151 (99.3)
Not performed	1 (0.7)
Trastuzumab therapy†	
Absent	141 (92.8)
Present	11 (7.2)
*ERBB2* CNA‡	
AMP	15 (9.9)
No AMP	137 (90.1)
HER2 status in the identical FFPE block analysis§	
IHC 3+	15 (9.9)
IHC 2+/FISH positive	6 (3.9)
IHC 2+/FISH negative	3 (2.0)
IHC 1+	37 (24.3)
IHC 0	91 (59.9)
HER2 status in the different FFPE block analysis‖	
IHC 3+	9 (15.8)
IHC 2+/FISH positive	5 (8.9)
IHC 2+/FISH negative	3 (5.2)
IHC 1+	8 (14.0)
IHC 0	32 (56.1)

*ERBB2*, erb-b2 receptor tyrosine kinase 2; CNA, copy number alteration; HER2, human epidermal growth factor receptor 2; GEJ, gastroesophageal junction; EBV, Epstein‒Barr virus; MSI, microsatellite instability; MSS, microsatellite stable; AMP, amplification; FFPE, formalin-fixed paraffin-embedded block; IHC, immunohistochemistry; FISH, fluorescence in situ hybridization

* According to the 8th edition of the TNM Classification of Malignant Tumours published by the Union for International Cancer Control

† Trastuzumab therapy was performed for unresectable metastatic disease

‡ The threshold for *ERBB2* AMP was defined as > 2.5-fold change in the targeted tumour-sequencing test

§ HER2 status was examined by IHC and FISH in the sections from FFPE blocks that were identical to those subjected to the targeted tumour sequencing test (*N* = 152)

‖ HER2 status was examined by IHC and FISH in the sections from FFPE blocks that were different from those subjected to the targeted tumour sequencing test (*N* = 57)

### Genomic profiling with the targeted tumour sequencing test

Genomic profiles in the primary tumour tissue were generated using a targeted tumour sequencing test containing 435 cancer-associated genes (CANCERPLEX; KEW Inc., Cambridge, MA), as described previously [14]. Briefly, genomic DNA was extracted from formalin-fixed, paraffin-embedded (FFPE) primary tumour tissues. We selected the FFPE block with the highest tumour content for each patient through a meticulous review of haematoxylin and eosin (H&E) stained slides archived subsequent to the pathological diagnosis. Referring to the H&E stained slides, tumour regions with > 20% purity of tumour cells on the unstained slides were macrodissected to enrich tumour content and subsequently subjected to DNA extraction. DNA fragment libraries were enriched for the coding regions and selected introns of the 435 genes and subjected to the Illumina MiSeq and NextSeq platforms (Illumina, San Diego, CA) for genomic sequencing with an average depth of 500×. Single-nucleotide variants (SNVs), short indels and somatic CNAs were called using a proprietary combination of callers. The cut-off value for SNVs and indels was a 10% allelic fraction, and those for AMP and deletion (DEL) were > 2.5-fold change (1.32 log2-fold change) and < 0.75-fold change (-0.42 log2-fold change), respectively. The designated CNA cut-off value was established by the developers of the analytical pipeline, and detection of *ERBB2* CNA was validated using cell lines and unspecified clinical samples [17]. Variants were filtered or flagged according to their technical quality (e.g., coverage, allelic fraction, number of supporting reads), presence in previously characterized normal samples, or presence/absence in the following databases: dbSNP, ExAC, COSMIC, ClinVar and KEW. We extracted information on *ERBB2* CNA and gene alterations in the following HER2 DPs by referring to cBioPortal [18]: RTK signalling (*EGFR*, *ERBB2*, *ERBB3*, *ERBB4*, *FGFR1*, *FGFR2*, *KDR*, *KIT*, *MET*, *PDGFRA* and *PDGFRB*), RAS-RAF-MEK signalling (*BRAF*, *HRAS*, *MAP2K1*, *MAP2K2*, *MAP2K4*, *MAP3K1*, *MAPK1*, *MAPK3*, *MAPK4*, *MAPK7*, *NRAS* and *RAF1*), PI3K-AKT-mTOR signalling (*AKT1*, *FOXO1*, *MTOR*, *PIK3CA*, *PIK3R1*, *PIK3R2*, *PTEN*, *RHEB*, *RICTOR*, *RPTOR*, *TSC1* and *TSC2*) and cell cycle control (*CCNE1*, *CDK1*, *CDK6*, *CDKN1B*, *CDKN2A*, *CDKN2B*, *JAK1*, *JAK2*, *MYC*, *SRC* and *STAT3*). The pathogenicity of the extracted alterations was determined according to the ClinVar (https://www.ncbi.nlm.nih.gov/clinvar/) and OncoKB (https://www.oncokb.org/) databases. Alterations annotated as benign or likely benign in ClinVar and as neutral or likely neutral in OncoKB were excluded from analyses in this study. MSI was tested based on an extended locus panel. In addition to the Bethesda panel, a collection of 950 regions consisting of tandem repeats of one, two or three nucleotides with a minimum length of 10 bases was used. Tumours were also analysed for the presence of an Epstein‒Barr virus (EBV) sequence using the reference genome of NC_007605. The percentage of the total number of reads mapped to the viral genome was calculated, and samples were designated as positive based on empirical cut-offs of 0.0005% of reads that mapped to the EBV genome.

### Evaluation of HER2 status with IHC and FISH

HER2 status was examined with a diagnostic kit validated by the Japanese Ministry of Health, Labour and Welfare, according to the manufacturer’s instructions. We selected the FFPE blocks that were identical to those subjected to the targeted tumour sequencing test. Three serial 4-µm-thick sections were cut from FFPE blocks, which included tumour tissues, and assigned for haematoxylin-eosin staining, anti-HER2 staining and a negative control. The sections were immunohistochemically stained with an anti-HER2 monoclonal antibody (SV2-61γ; Nichirei Biosciences, Inc., Tokyo, Japan). HER2 expression was scored according to the following criteria: 0 for no staining or membrane staining in less than 10% of invasive tumour cells; 1 + for weak membrane staining in 10% or more of invasive tumour cells; 2 + for weak to moderate complete or basolateral membrane staining in 10% or more of invasive tumour cells; and 3 + for moderate to strong complete or basolateral membrane staining in 10% or more of invasive tumour cells. *ERBB2* amplification was evaluated by FISH in cases with IHC 2+. FISH was performed with a PathVysion HER2 DNA probe kit (Abbott Japan, Tokyo, Japan), and gene amplification was evaluated according to the fluorescence signal ratio of *ERBB2* to chromosome enumeration probe 17 (CEP17). FISH positivity with gene amplification was defined as an *ERBB2*-to-CEP17 ratio of 2.0 or greater. HER2 positivity was defined as IHC 3 + or IHC 2+/FISH positivity, and HER2 negativity was defined as IHC 2+/FISH negativity, IHC 1 + or 0, according to the ToGA study [8]. In addition, we retrospectively reviewed HER2 status information in patients whose HER2 status was evaluated via clinical testing using sections derived from FFPE blocks, which differed from those used for the targeted tumour sequencing test.

### Outcomes of trastuzumab therapy for unresectable metastatic gastric cancer

We retrospectively reviewed clinical information on trastuzumab therapy for unresectable metastatic GC in 11 patients. Radiographic tumour assessments were performed using enhanced computed tomography of the chest, abdomen and pelvis. Treatment response was evaluated according to Response Evaluation Criteria in Solid Tumours (RECIST), version 1.1 [19]. The objective response rate (ORR) and disease control rate (DCR) were assessed using the best overall response in measurable target lesions. ORR and DCR were defined as the proportion of patients who achieved complete response (CR) or partial response (PR) and the proportion who achieved CR, PR or stable disease (SD), respectively. Progression-free survival (PFS) was defined as the period from the day of starting trastuzumab therapy to the day of disease progression or death from any cause.

### Statistical analysis

Differences in continuous variables were assessed using the Mann‒Whitney *U* test for dichotomous groups and the Kruskal‒Wallis test for polychotomous groups. Differences in categorical variables between groups were assessed using Fisher’s exact test. Concordance between *ERBB2* CNA and HER2 status was evaluated using positive percent agreement (PPA), negative percent agreement (NPA), overall percent agreement (OPA) and Cohen’s kappa with 95% confidence intervals (CIs). The relative strength of agreement was assessed according to the following criteria: kappa > 0.8, almost perfect; 0.6 < kappa ≤ 0.8, substantial; 0.4 < kappa ≤ 0.6, moderate; 0.2 < kappa ≤ 0.4, fair; 0 ≤ kappa ≤ 0.2, slight; and kappa < 0, poor [20]. We estimated cumulative PFS rates using the Kaplan‒Meier method, and differences between the groups were assessed using the log-rank test. A *P* value less than 0.05 (two-tailed) was considered to indicate statistical significance. All statistical analyses were performed using the R programming language and environment (version 4.3.1; http://www.r-project.org).

## Results

### Differences in *ERBB2* CNA according to HER2 status

**Fig. 1:**
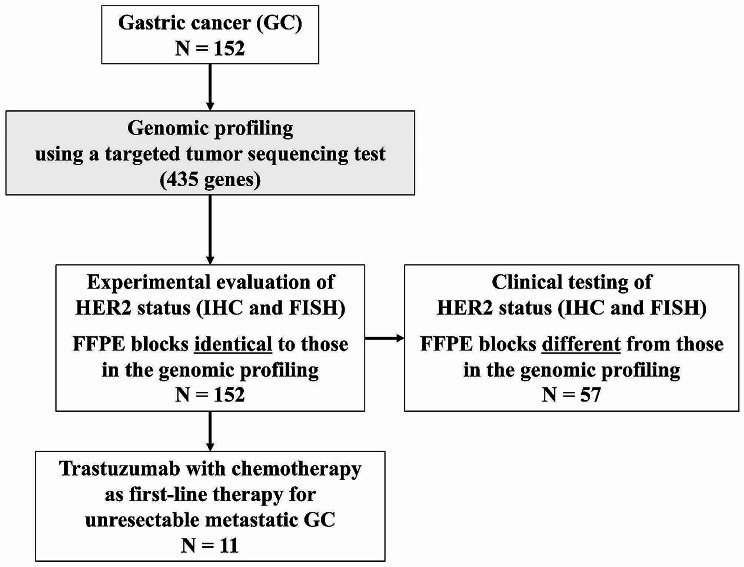
Flow diagram of the patients included in analyses

**Fig. 2:**
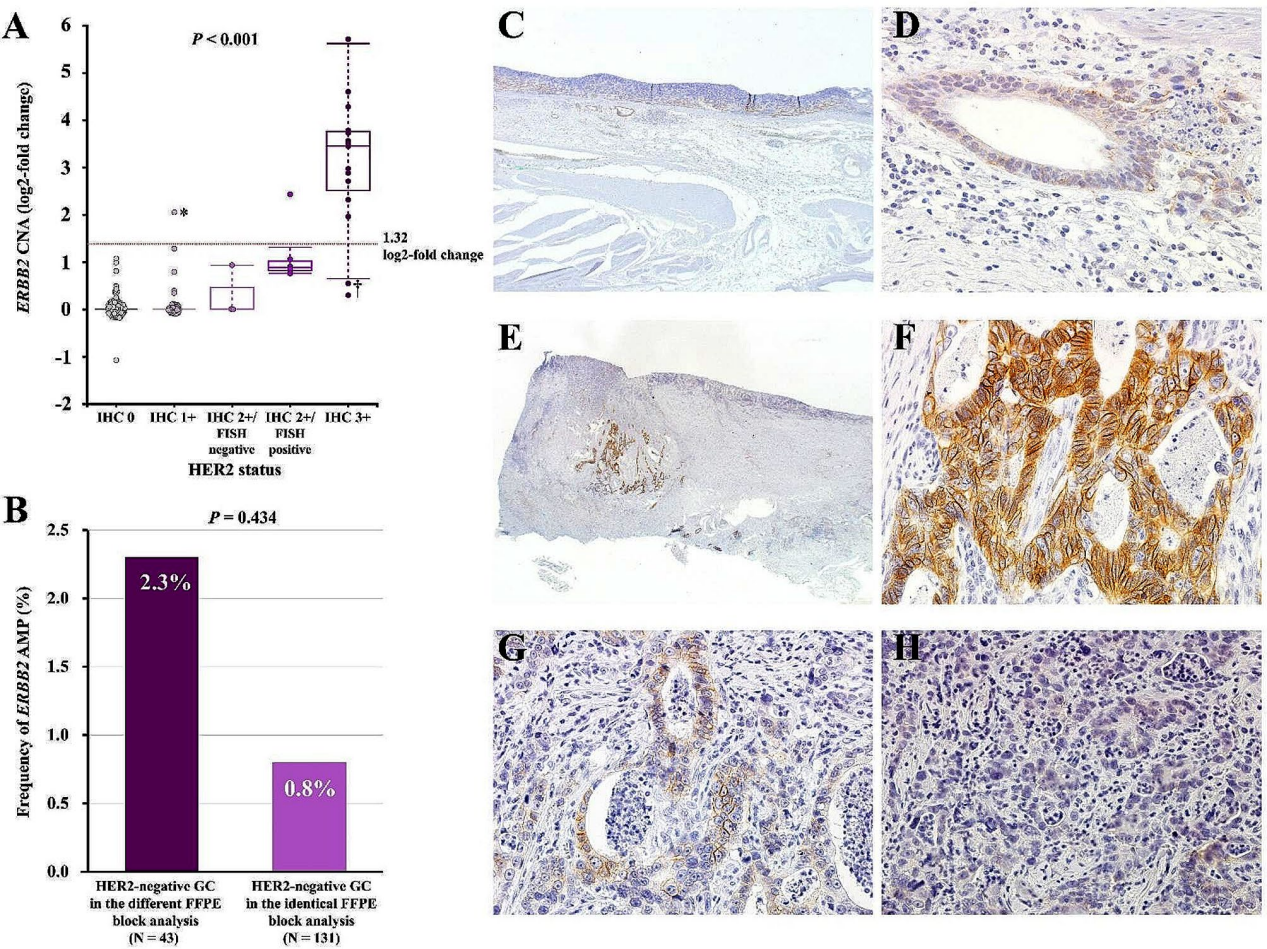
Association between *ERBB2* CNA and HER2 status. *ERBB2* CNA (log2-fold change) was evaluated by a targeted tumour sequencing test according to HER2 status (**A**). Frequency of *ERBB2* AMP detected by the targeted tumour sequencing test among HER2-negative GCs in identical and different FFPE block analyses (**B**). HER2-IHC in tumours with *ERBB2* AMP (4.14-fold change) and HER2 negativity with IHC 1 + indicated by asterisks (*) in **A** (**C**: overview, original magnification × 20, **D**: weak membrane staining, × 400). HER2-IHC in tumours with no *ERBB2* AMP (1.46-fold change) and HER2 positivity with IHC 3 + including heterogeneous HER2 staining indicated by the dagger symbol (†) in **A** (**E**: overview, original magnification × 12.5, **F**: strong complete or basolateral membrane staining, × 400, **G**: weak to moderate complete or basolateral membrane staining × 400, **H**: no staining × 400). *ERBB2*, erb-b2 receptor tyrosine kinase 2; CNA, copy number alteration; HER2, human epidermal growth factor receptor 2; AMP, amplification; FFPE, formalin-fixed paraffin-embedded block; IHC, immunohistochemistry

A targeted tumour sequencing test detected *ERBB2* CNA in 152 tumours with a median fold change (range) of 1.0 (0.5–52.4), corresponding to a log2-fold change (range) of 0 (-1.07–5.71). We evaluated HER2 status by IHC and FISH in tumour tissue sections derived from FFPE blocks that were identical to those subjected to the targeted tumour sequencing test (identical FFPE block analysis, Fig. 1). HER2 positivity (IHC 3 + or IHC 2+/FISH positivity) was detected in the tumours of 21 patients (13.8%), including 15 (9.9%) with IHC 3 + and 6 (3.9%) with IHC 2+/FISH positive tumours. HER2 negativity (IHC 2+/FISH negativity, IHC 1 + or IHC 0) was detected in the tumours of the remaining 131 patients (86.2%), including 3 (2.0%) IHC 2+/FISH negative, 37 (24.3%) IHC 1 + and 91 (59.9%) IHC 0 tumours (Table 1). We compared the *ERBB2* CNA according to HER2 status (Fig. 2A). The median log2-fold changes (range) for *ERBB2* CNA in IHC 3+, IHC 2+/FISH positive, IHC 2+/FISH negative, IHC 1 + and IHC 0 tumours were 3.45 (0.26–5.71), 0.85 (0.77–2.43), 0 (0–0.93), 0 (0–2.05) and 0 (-1.07–1.08), respectively. The difference in the fold change for *ERBB2* CNA according to HER2 status was statistically significant (*P* < 0.001).

### Concordance between *ERBB2* CNA evaluated by the targeted tumour sequencing test and HER2 status

The targeted tumour sequencing test detected *ERBB2* AMP (> 2.5-fold change) in the tumours of 15 patients (9.9%). Fourteen of the 21 patients with HER2-positive tumours had *ERBB2* AMP, and the PPA was calculated to be 66.7% in the identical FFPE block analysis (Table 2). Among 131 patients with HER2-negative tumours, no *ERBB2* AMP was detected in the tumours of 130 patients, and the NPA was calculated to be 99.2% in the identical FFPE block analysis. Among the 152 patients, concordant and discordant results between *ERBB2* CNA and HER2 status were obtained for 144 (concordant group) and 8 (discordant group), respectively. Thus, the OPA was calculated to be 94.7%, and there was substantial agreement with Cohen’s kappa of 0.75 (95% CI, 0.58–0.91) in the identical FFPE block analysis (Table 2). Among the 152 patients, 57 had HER2 status information evaluated via clinical testing using sections derived from FFPE blocks, which differed from those used for the targeted tumour sequencing test (Fig. 1). We assessed the concordance between *ERBB2* CNA and HER2 status evaluated using different FFPE blocks (different FFPE block analyses, Fig. 1; Table 2). Among 57 patients, 14 (24.6%) had HER2-positive tumours, including 9 (15.8%) with IHC 3 + and 5 (8.9%) with IHC 2+/FISH positive tumours. The remaining 43 patients (75.4%) had HER2-negative tumours, including 3 (5.3%) with IHC 2+/FISH negative tumours, 8 (14.0%) with IHC 1 + tumours and 32 (56.1%) with IHC 0 tumours. The PPA and NPA in the different FFPE block analyses were 50.0% and 97.7%, respectively. The OPA in the different FFPE block analyses was 86.0%, which was significantly lower than that in the identical FFPE block analysis (*P* = 0.043). There was moderate agreement with Cohen’s kappa of 0.56 (95% CI, 0.29–0.82) in the different FFPE block analyses. The frequency of *ERBB2* AMP detected by the targeted tumour sequencing test in HER2-negative GC was 0.8% (1 of 131 patients) in the identical FFPE block analysis and 2.3% (1 of 43 patients) in the different FFPE block analysis (*P* = 0.434, Fig. 2B).

**Table 2:** Concordance between *ERBB2* CNA evaluated by the targeted tumour sequencing test and HER2 status

		Number of patients				Number of patients			*P* value
		HER2 status in the identical FFPE block analysis†				HER2 status in the different FFPE block analysis‡			
		Positive	Negative	Total		Positive	Negative	Total	
*ERBB2* CNA evaluated by the targeted tumour sequencing test*	AMP	14	1	15		7	1	8	NE
	No AMP	7	130	137		7	42	49	
	Total	21	131	152		14	43	57	
	Positive percent agreement (95% CI)	66.7% (43.0–85.4%)				50.0% (23.0–77.0%)			0.483
	Negative percent agreement (95% CI)	99.2% (95.8–99.9%)				97.7% (87.7–99.9%)			0.434
	Overall percent agreement (95% CI)	94.7% (90.0–97.7%)				86.0% (74.2–93.7%)			0.043
	Cohen’s kappa (95% CI)	0.75 (0.58–0.91)				0.56 (0.29–0.82)			NE

*ERBB2*, erb-b2 receptor tyrosine kinase 2; CNA, copy number alteration; HER2, human epidermal growth factor receptor 2; AMP, amplification; FFPE, formalin-fixed paraffin-embedded block; CI, confidence interval; NE, not evaluated

* The threshold for *ERBB2* amplification was defined as > 2.5-fold change (1.32 log2-fold change) in the targeted tumour sequencing test

† HER2 status in the sections from FFPE blocks that were identical to those subjected to the targeted tumour sequencing test (*N* = 152)

‡ HER2 status in the sections from FFPE blocks that were different from those subjected to the targeted tumour sequencing test (*N* = 57)

### Clinicopathological features associated with discordance between ERBB2 CNA and HER2 status

The clinicopathological features in patients with discordant results between *ERBB2* CNA and HER2 status (discordant group, *N* = 8) were compared to those in patients with concordant results (concordant group, *N* = 144) via identical FFPE block analysis (Table 3). Regarding HER2 status, the proportion of IHC 2+/FISH-positive tumours in the discordant group was significantly higher than that in the concordant group (62.5% vs. 0.7%, *P* < 0.001). The other clinicopathological features did not differ significantly between the groups (Table 3). The details of *ERBB2* CNA and HER2 status in the discordant group are shown in Table 4. Tumours with *ERBB2* AMP (4.14-fold change) and HER2 negativity were IHC 1+ (Fig. 2C and D). On the other hand, tumours with no *ERBB2* AMP (1.46-fold change) and HER2 positivity were IHC 3+, with heterogeneous HER2 staining consisting of weak to moderate complete or basolateral membrane staining and no staining (Fig. 2E and H).

**Table 3:** Clinicopathological features and the concordance between *ERBB2* CNA and HER2 status

Variable	Number of patients (%)		*P * value
	Concordant‡ (*N* = 144)	Discordant§ (*N* = 8)	
Age (years)			
< 68	70 (48.6)	6 (75.0)	0.276
≥ 68	74 (51.4)	2 (25.0)	
Sex			
Male	102 (70.8)	8 (100.0)	0.107
Female	42 (29.2)	0 (0.0)	
Location			
GEJ or Cardia	26 (18.1)	1 (12.5)	0.891
Body or Fundus	54 (37.5)	4 (50.0)	
Antrum	64 (44.4)	3 (37.5)	
Lauren classification			
Intestinal	82 (56.9)	8 (100.0)	0.215
Diffuse	40 (43.1)	0 (0.0)	
Mixed	9 (6.3)	0 (0.0)	
Indeterminate	13 (9.0)	0 (0.0)	
T classification*			
T1	4 (2.8)	0 (0.0)	> 0.999
T2–4	140 (97.2)	8 (100.0)	
N classification*			
N0	40 (27.8)	1 (12.5)	0.683
N1–3	104 (72.2)	7 (87.5)	
M classification*			
M0	115 (79.9)	8 (100.0)	0.354
M1	29 (20.1)	0 (0.0)	
Lymphatic invasion†			
Absent	41 (28.7)	3 (37.5)	0.692
Present	102 (71.3)	5 (62.5)	
Venous invasion†			
Absent	64 (44.8)	5 (62.5)	0.470
Present	79 (55.2)	3 (37.5)	
EBV infection			
Absent	141 (97.9)	7 (87.5)	0.196
Present	3 (2.1)	1 (12.5)	
MSI status			
MSI-high	13 (9.0)	0 (0.0)	> 0.999
MSI-low or MSS	131 (91.0)	8 (100.0)	
HER2 status			
IHC 3+	13 (9.0)	2 (25.0)	< 0.001
IHC 2+/FISH positive	1 (0.7)	5 (62.5)	
Negative	130 (90.3)	1 (12.5)	

*ERBB2*, erb-b2 receptor tyrosine kinase 2; HER2, human epidermal growth factor receptor 2; CNA, copy number alteration; GEJ, gastroesophageal junction; EBV, Epstein‒Barr virus; MSI, microsatellite instability; MSS, microsatellite stable; IHC, immunohistochemistry; FISH, fluorescence in situ hybridization

* According to the 8th edition of the TNM Classification of Malignant Tumours published by the Union for International Cancer Control

† One patient with no information on lymphatic invasion and venous invasion was excluded

‡ The concordant group included patients with *ERBB2* amplification and HER2 positivity or patients with *ERBB2* no amplification and HER2 negativity

§ The discordant group included patients with *ERBB2* amplification and HER2 negativity or patients with *ERBB2* no amplification and HER2 positivity

**Table 4:** Detail of discordant findings of *ERBB2* CNA and HER2 status

Patient	*ERBB2* CNA (fold change)	HER2 status	IHC	FISH
88*	AMP (4.14)	Negative	1+	NA
69†	No AMP (1.46)	Positive	3+	NA
134	No AMP (1.23)	Positive	3+	NA
49	No AMP (1.75)	Positive	2+	Positive
70	No AMP (1.86)	Positive	2+	Positive
75	No AMP (2.08)	Positive	2+	Positive
93	No AMP (1.83)	Positive	2+	Positive
111	No AMP (1.69)	Positive	2+	Positive

* Identical to the patient indicated by the asterisk symbol in Fig. 2A

† Identical to the patient indicated by the dagger symbol in Fig. 2A

*ERBB2*, erb-b2 receptor tyrosine kinase 2; CNA, copy number alteration; HER2, human epidermal growth factor receptor 2; IHC, immunohistochemistry; FISH, fluorescence in situ hybridization; AMP, amplification; NA, not analysed

### Tumour response to anti-HER2 therapy and genetic alterations detected by the targeted tumour sequencing test

**Fig. 3:**
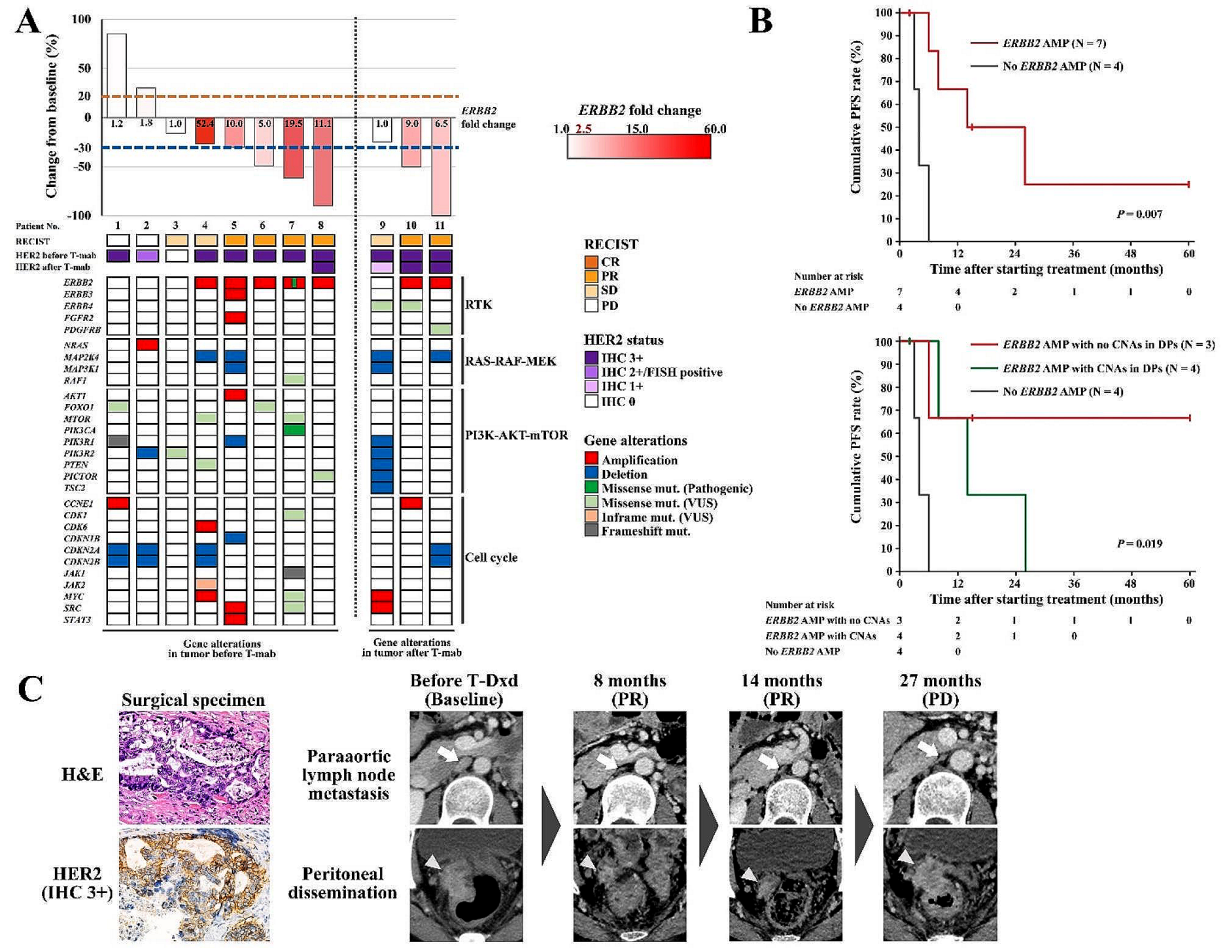
Treatment response to anti-HER2 therapies for unresectable metastatic gastric cancer in relation to *ERBB2* CNA and genetic alterations in HER2 DPs. Waterfall plot of the best overall response to trastuzumab therapy with HER2 status and genetic alterations detected by the targeted tumour sequencing test (**A**). Kaplan-Meier survival curves stratified by *ERBB2* CNA (top) and *ERBB2* CNA with CNAs in HER2 DPs (bottom) for PFS after starting trastuzumab therapy (**B**). H&E staining and HER2 IHC in primary tumour tissue of the surgical specimen (original magnification × 400) and CT images of paraaortic lymph node metastasis (arrow) and peritoneal dissemination (arrowhead) after curative gastrectomy in patients with a durable response to T-Dxd as fourth-line therapy lasting 27 months, indicated as No. 4 in **A** (**C**). HER2, human epidermal growth factor receptor 2; *ERBB2*, erb-b2 receptor tyrosine kinase 2; CNAs, copy number alterations; T-mab, trastuzumab; DPs, downstream pathways; PFS, progression-free survival; H&E, haematoxylin and eosin; IHC, immunohistochemistry; CT, computed tomography; T-Dxd, trastuzumab deruxtecan

Among the 152 patients, trastuzumab combined with chemotherapy was administered to 11 patients with unresectable metastatic GC. The patient and treatment characteristics are detailed in Additional file 1. Tumour samples subjected to the targeted tumour sequencing test were obtained before and after trastuzumab therapy from 8 to 3 patients, respectively. A waterfall plot of the best overall response for each patient with genomic alterations is shown in Fig. 3A. Among 7 patients with tumours showing *ERBB2* AMP and IHC 3+ (Nos. 4–8, 10 and 11), 6 patients (Nos. 5–8, 10 and 11) achieved PR on trastuzumab therapy. On the other hand, 2 patients (Nos. 1 and 2) with no *ERBB2* AMP and IHC 3 + or IHC 2+/FISH positive (discordancy) tumours showed no response to trastuzumab therapy, which was defined as PD. The ORR for trastuzumab therapy in patients with *ERBB2* AMP was 85.7%, and that in patients with no *ERBB2* AMP was 0% (*P* = 0.015, Table S1). Moreover, PFS in patients with *ERBB2* AMP was significantly better than that in patients with no *ERBB2* AMP (median 14 months vs. 4 months, *P* = 0.007; Fig. 3B). Genetic alterations in HER2 DPs were detected in all 11 patients, including mutations (missense, in-frame or frameshift) in 4, CNAs in 2 and both mutations and CNAs in 5 (Fig. 3A). Three patients (Nos. 6–8) with *ERBB2* AMP and no CNAs in tumour samples before trastuzumab therapy had a remarkable response to the therapy. In contrast, treatment response was diminished in two patients (Nos. 4 and 5) who had *ERBB2* AMP with concomitant CNAs in HER2 DPs. The median PFS was not reached in patients with *ERBB2* AMP and no CNAs in HER2 DPs; it was 14 months in patients with *ERBB2* AMP and CNA, and 4 months in patients with no *ERBB2* AMP (*P* = 0.019, Fig. 3B). One patient (No. 4 in Fig. 3A) who had *ERBB2* AMP with concomitant CNAs in HER2 DPs achieved durable response to trastuzumab deruxtecan (T-Dxd) lasting 27 months (Fig. 3C). This patient was administered trastuzumab combined with S-1 plus cisplatin as a first-line therapy for paraaortic lymph node metastasis after curative gastrectomy according to HER2 positivity with IHC 3 + confirmed in the surgical specimen. After trastuzumab therapy with the best overall response of SD and subsequent ramucirumab combined with paclitaxel as second-line therapy and nivolumab as third-line therapy, T-Dxd was administered as fourth-line therapy for disease progression involving paraaortic lymph node metastasis and peritoneal dissemination. PR in the targeted lesions was achieved after 8 months of treatment, and T-Dxd therapy was continued until disease progression involving peritoneal dissemination at 27 months (Fig. 3C). The targeted tumour sequencing test of the sample before trastuzumab therapy revealed *ERBB2* AMP (52.4-fold change) with concomitant *CDK6* and *MYC* AMP, *CDKN2A* and *CDKN2B* DEL, *MAP2K4* DEL, *MTOR* and *PTEN* missense mutation and *JAK2* inflame mutation (No. 4 in Fig. 3A).

## Discussion

Precision medicine in GC is presently in a nascent stage, and the clinical implications surrounding utilization of a targeted tumour sequencing test for HER2-positive GC, one of the actionable subtypes eligible for molecularly targeted therapy, remain unclear. This study illuminates the high accuracy of the targeted tumour sequencing test in determining the HER2 status of GC. In addition, the concordance between *ERBB2* CNA identified through the targeted tumour sequencing test and HER2 status assessed by IHC and FISH was notably diminished in different block analyses compared to identical block analyses. Furthermore, through the targeted tumour sequencing test, *ERBB2* CNA emerged as a promising metric indicative of response to trastuzumab therapy and progression-free survival. CNAs in HER2 DPs may also play a role in conferring resistance to trastuzumab therapy. Collectively, our findings suggest that use of a targeted tumour sequencing test may serve as a valuable tool for characterizing the molecular subtype of GC and optimizing molecularly targeted therapies for primary management of unresectable metastatic GC.

The robustness of targeted tumour sequencing tests in determining HER2-positive GC constitutes a pivotal facet of precision medicine for GC. The OPA between *ERBB2* CNA identified through Foundation One CDx, a globally recognized targeted tumour sequencing test, and the HER2 status assessed by IHC or ISH, which serves as a companion diagnostic tool for anti-HER2 therapy, is reported to be 87.5% [21]. MSK-IMPACT, devised by the Memorial Sloan Kettering Cancer Center, exhibited a concordance rate of 93.7%, with a positive predictive value of 90% and a negative predictive value of 96.9% in the context of similar evaluations [22]. These investigations predominantly encompassed cohorts of GC patients in the United States. In this investigation, we demonstrated a high OPA and Cohen’s kappa between *ERBB2* CNA and HER2 status within a cohort of Japanese GC patients. The targeted tumour sequencing test for discerning *ERBB2* CNA shows reliability in confirming that HER2-positive GC is a robust indication for anti-HER2 therapy, regardless of geographic region.

In this study, the NPA between *ERBB2* CNA identified through the targeted tumour sequencing test and HER2 status assessed by IHC and FISH was as high as 99.2%. Similarly, the NPA was reported to be 98.4% in an investigation using Foundation One CDx [21]. These findings suggest that when the panel test identifies *ERBB2* AMP, the patient can be definitively confirmed to have HER2-positive GC. Conversely, the PPA in this study was lower than the NPA, similar to the findings of the investigation using Foundation One CDx [21]. A lower PPA indicates a greater proportion of false negatives. As shown in Table 4, the majority of patients exhibiting false negativity for *ERBB2* CNA had HER2-positive GC with IHC 2+/FISH positivity. Conspicuous heterogeneity in IHC scores for the tumour tissue was found for patients who presented with an HER2 status of IHC 3 + but with a false negative for *ERBB2* CNA. After subgroup analysis of the ToGA study, no statistically significant additive effect of trastuzumab was observed in HER2-positive GC with IHC 2+/FISH positivity [8]. Heterogeneity in HER2 IHC scores has been shown to be associated with poor response to trastuzumab therapy [23, 24]. Patients with false-negative results with Foundation One CDx had a shorter time to treatment discontinuation and overall survival following first-line trastuzumab therapy [21]. The *ERBB2* CNA cut-off value for the targeted tumour sequencing test employed in this study was established by the developers of the analytical pipeline, and detection of *ERBB2* CNA was validated using cell lines and unspecified clinical samples [17]. Although a reduction in the cut-off value of the test might identify false-negative cases, such adjustment is unsuitable for selection of candidates for trastuzumab therapy. In the present study, compared with no *ERBB2* AMP, *ERBB2* AMP was associated with better PFS and a higher ORR for trastuzumab therapy. Furthermore, several reports attest to the association of *ERBB2* AMP, as determined through copy number analysis using NGS, with more favourable prognosis in trastuzumab therapy [22, 25–27]. Assessment of *ERBB2* CNA via a targeted tumour sequencing test is advantageous for identifying patients likely to exhibit a favourable response to trastuzumab therapy. However, the optimal cut-off value of *ERBB2* CNA for predicting prognosis in patients receiving trastuzumab therapy remains undetermined. Previous studies have demonstrated that elevated continuous copy numbers of *ERBB2* correlate significantly with extended PFS [25, 26], and higher cut-off values of *ERBB2* CNA, compared to those employed in the current study, have proven advantageous for prognostic categorization in patients undergoing trastuzumab therapy [26, 27]. Further exploration aimed at delineating the optimal cut-off values capable of predicting the efficacy of trastuzumab therapy specifically in patients with GC will advance anti-HER2 therapy for unresectable metastatic disease.

Selection of FFPE blocks for molecular testing is critical in GC, which displays extensive intratumoral heterogeneity of molecular backgrounds [28, 29]. In this investigation, we also examined the concordance between *ERBB2* CNA and HER2 status in 57 patients whose HER2 status was evaluated using a different FFPE block as a routine clinical practice. The OPA between the *ERBB2* CNA and HER2 status in the different FFPE blocks was lower than that in the identical FFPE blocks. A low PPA of 56.2% was also reported in the examination using Foundation One CDx, which was attributed to the fact that IHC and ISH were performed in various pathology labs using different specimens from the targeted tumour sequencing test [21]. This means that HER2 expression differs between blocks due to the intratumoral heterogeneity of the molecular background in GC. A patient who has been treated for HER2-negative GC with negative prior IHC and FISH results and shows *ERBB2* AMP in a subsequent targeted tumour sequencing test may derive a benefit from additional anti-HER2 therapy options. However, there was no significant difference in the frequency of *ERBB2* AMP detection among HER2-negative tumours between identical FFPE blocks and different FFPE blocks in this study. In other words, even if an FFPE block different from that used for HER2 testing is selected as the specimen for the targeted tumour sequencing test, there is a small possibility of providing an opportunity for anti-HER2 therapy to those who were previously treated as HER2 negative. In selection of specimens for a targeted tumour sequencing test, there is no higher priority than FFPE block, which should have a high proportion of tumour content allowing sufficient tumour-derived DNA for extraction [30].

Molecular mechanisms of resistance to trastuzumab therapy include AMP of receptor tyrosine kinases other than HER2 and genetic alterations within HER2 DPs, such as the PI3K-AKT-mTOR and MAPK pathways [31–34]. NGS-based tumour sequencing tests target multiple genes, facilitating concurrent evaluation of genetic alterations within these pathways [35]. In this study, patients who displayed CNAs in HER2 DPs exhibited a diminished response rate and poorer PFS to trastuzumab therapy. Several previous studies have shown that mutations and CNAs in HER2 DPs identified through NGS in GC are associated with resistance to trastuzumab therapy, corroborating the findings of this investigation [22, 26, 36, 37]. Regrettably, a definitive therapeutic strategy for HER2-positive GC patients with genetic alterations in HER2 DPs remains elusive. A potentially effective anticancer agent for trastuzumab-resistant GC is T-Dxd, an antibody–drug conjugate consisting of a humanized monoclonal anti-HER2 antibody, an enzymatically cleavable peptide-based linker, and a novel cytotoxic topoisomerase I inhibitor [38]. The DESTINY-Gastric01 trial demonstrated a significant prognostic advantage of T-Dxd over standard chemotherapy as third-line or subsequent therapy for HER2-positive GC that progressed despite trastuzumab therapy [39]. As highlighted in Fig. 3C, a patient harbouring a genetic alteration in HER2 DPs was treated with T-Dxd as the fourth line of therapy after trastuzumab, which resulted in a durable therapeutic response and prolonged treatment duration. The ongoing DESTINY-Gastric03 trial is evaluating the efficacy and safety of T-Dxd in combination with anticancer agents and immune checkpoint inhibitors as first-line therapy [38]. Thus, T-Dxd, which has considerable therapeutic efficacy independent of potential trastuzumab resistance, holds promise as a pivotal anticancer agent for HER2-positive GC.

This study has several limitations. The targeted tumour sequencing test utilized in this study lacks approval for clinical utilization worldwide. The real-world efficacy in discerning HER2-positive GC via clinically available sequencing tests, such as Foundation One CDx or OncoGuide NCC Oncopanel, should be evaluated using high-quality clinical and sequencing datasets in Japan [40]. The number of patients who received trastuzumab therapy among the enrolled patients was only 11. The genetic alterations identified in three patients after trastuzumab therapy could not be distinguished based on whether they were present prior to therapy or were acquired afterwards. Furthermore, we have not demonstrated that genetic alterations in HER2 DPs are involved in therapeutic resistance to trastuzumab via the in vitro or in vivo experiments. We could not draw a definite conclusion that *ERBB2* AMP or genetic alterations in HER2 DPs are associated with trastuzumab treatment response or patient prognosis, and further investigations and validation studies are necessary. Nonetheless, we believe that the findings of this study can serve as a basis for further advancements in precision medicine in the field of GC.

## Conclusions

A targeted tumour sequencing test is a reliable modality for detecting HER2-positive GC. *ERBB2* AMP and concomitant genetic alterations detected through the targeted tumour sequencing test are potential indicators for assessing treatment response to trastuzumab therapy. The targeted tumour sequencing test has emerged as a plausible candidate for companion diagnostics to determine the indication for anti-HER2 therapy in the era of precision medicine for GC.

### Electronic supplementary material

Below is the link to the electronic supplementary material.

Supplementary Material 1

Supplementary Material 2

## Data Availability

Of the sequencing data from 152 patients utilized in this study, 130 were derived from our previous research, while 22 were newly acquired for this investigation. The sequencing data have not been uploaded to public databases, as consent from the study participants to deposit the data was not obtained. The datasets including the sequencing data used in this study are available from the corresponding author upon reasonable request.
