# Supplementary material for: Clinical application of targeted tumour sequencing tests for detecting ERBB2 amplification and optimizing anti-HER2 therapy in gastric cancer
Source: BMC Cancer. 2024 Jun 11;24:719. doi: 10.1186/s12885-024-12482-5 (PMC11167924; doi:10.1186/s12885-024-12482-5)
Supplement: Supplementary file 1 — Supplementary Material 1 [file 12885_2024_12482_MOESM1_ESM.docx]

| **Table S1.** Patient and treatment characteristics in trastuzumab therapy (N = 11) | | | |  |
| --- | --- | --- | --- | --- |
|  | Number of patients (%) | | |  |
| Characteristics | Total | *ERBB2* AMP  (N = 7) | No *ERBB2* AMP  (N = 4) | *P* value |
| Sex |  |  |  |  |
| Male | 7 (63.6) | 4 (57.1) | 3 (75.0) | > 0.999 |
| Female | 4 (36.4) | 3 (42.9) | 1 (25.0) |  |
| Age (years) |  |  |  |  |
| ≥ 65 years | 7 (63.6) | 3 (42.9) | 4 (100) | 0.194 |
| < 65 years | 4 (36.4) | 4 (57.1) | 0 (0) |  |
| Liver metastasis* |  |  |  |  |
| Absent | 4 (36.4) | 3 (42.9) | 1 (25.0) | > 0.999 |
| Present | 7 (63.6) | 4 (57.1) | 3 (75.0) |  |
| Lung metastasis* |  |  |  |  |
| Absent | 7 (63.6) | 4 (57.1) | 3 (75.0) | > 0.999 |
| Present | 4 (36.4) | 3 (42.9) | 1 (25.0) |  |
| Lymph node metastasis* |  |  |  |  |
| Absent | 6 (54.4) | 3 (42.9) | 3 (75.0) | 0.545 |
| Present | 5 (45.5) | 4 (57.1) | 1 (25.0) |  |
| Peritoneal dissemination* |  |  |  |  |
| Absent | 11 (100) | 7 (100) | 4 (100) | NA |
| Present | 0 (0) | 0 (0) | 0 (0) |  |
| Number of metastatic lesions* |  |  |  |  |
| 1 | 7 (63.6) | 4 (57.1) | 3 (75.0) | > 0.999 |
| 2 | 3 (27.3) | 2 (28.6) | 1 (25.0) |  |
| 3 | 1 (9.1) | 1 (14.3) | 0 (0) |  |
| Combined chemotherapy |  |  |  |  |
| Capecitabine plus cisplatin | 5 (45.5) | 2 (28.6) | 3 (75.0) | 0.697 |
| Capecitabine plus oxaliplatin | 1 (9.1) | 1 (14.3) | 0 (0) |  |
| S-1 plus cisplatin | 5 (45.5) | 4 (57.1) | 1 (25.0) |  |
| Best overall response† |  |  |  |  |
| CR | 0 (0) | 0 (0) | 0 (0) | 0.015 |
| PR | 6 (54.5) | 6 (85.7) | 0 (0) |  |
| SD | 3 (27.3) | 1 (14.3) | 2 (50.0) |  |
| PD | 2 (18.2) | 0 (0) | 2 (50.0) |  |
| Objective response (CR or PR) | 6 (54.4) | 6 (85.7) | 0 (0) | 0.015 |
| Disease control (CR, PR or SD) | 9 (81.8) | 7 (100) | 2 (50.0) | 0.109 |
| HER2, human epidermal growth factor receptor 2; *ERBB2*, erb-b2 receptor tyrosine kinase 2; AMP, amplification; NA, not available. | | | | |

* Initial metastatic site.

† Treatment response was evaluated by RECIST ver. 1.1.
